# Integer Versus Fractional Order SEIR Deterministic and Stochastic Models of Measles

**DOI:** 10.3390/ijerph17062014

**Published:** 2020-03-18

**Authors:** Md Rafiul Islam, Angela Peace, Daniel Medina, Tamer Oraby

**Affiliations:** 1 Department of Mathematics and Statistics, Texas Tech University, 2500 Broadway, Lubbock, TX 79409, USA; a.peace@ttu.edu; 2 School of Mathematical and Statistical Sciences, The University of Texas Rio Grande Valley, 1201 W. University Drive, Edinburg, TX 78539, USA; dpmedin@gmail.com (D.M.); tamer.oraby@utrgv.edu (T.O.)

**Keywords:** fractional SEIR stochastic model, caputo fractional order differential equations, measles, parameter estimation

## Abstract

In this paper, we compare the performance between systems of ordinary and (Caputo) fractional differential equations depicting the susceptible-exposed-infectious-recovered (SEIR) models of diseases. In order to understand the origins of both approaches as mean-field approximations of integer and fractional stochastic processes, we introduce the fractional differential equations (FDEs) as approximations of some type of fractional nonlinear birth and death processes. Then, we examine validity of the two approaches against empirical courses of epidemics; we fit both of them to case counts of three measles epidemics that occurred during the pre-vaccination era in three different locations. While ordinary differential equations (ODEs) are commonly used to model epidemics, FDEs are more flexible in fitting empirical data and theoretically offer improved model predictions. The question arises whether, in practice, the benefits of using FDEs over ODEs outweigh the added computational complexities. While important differences in transient dynamics were observed, the FDE only outperformed the ODE in one of out three data sets. In general, FDE modeling approaches may be worth it in situations with large refined data sets and good numerical algorithms.

## 1. Introduction

Modeling the spread of infectious diseases before the introduction of vaccines, as well as the validation of these models, has been widely studied since the works of Reference [1,2,3,4,5,6,7,8,9,10]. See Bailey [11] and Anderson [12] for more details about the history of disease modeling. Deterministic models using ordinary differential equations (ODEs) have received great attention [12,13,14,15,16] and wide assimilation by health sciences [17]. Other deterministic models, such as difference equations, are also used to model the spread of diseases; for instance, see Fisman et al. [18]. However, fractional differential equations (FDEs) have been used in the last decade to model the course of epidemics [19,20,21,22,23,24].

Fractional differential equations are usually used to involve the memory of the process in the dynamics of the systems. There is more than one type of fractional order derivative, most notably, Caputo, Grünwald-Letnikov, and Riemann-Liouville [25]. Here, we study the Caputo fractional order derivative. Integer order derivatives of ordinary differential equations are special cases of fractional order derivatives. It was noted in more than one paper, e.g., Reference [26], that FDEs give a better depiction of the courses of epidemics and natural phenomena than ODEs. Few researchers have also fitted their FDE models to data, e.g., Reference [26,27]. This motivated us to compare systems of ODEs and FDEs by fitting them to some actual epidemic data.

Measles is a marker disease for virological, epidemiological, clinical, statistical, geographical, mathematical, and humanitarian reasons [28] (pp. 16–21). Mathematical modeling of measles epidemics dates back as far as 1888 by D’Enko and then by Hamer; see Haggett [28] (p. 19). Regularity and a large number of cases of measles epidemics with major peaks in the pre-vaccination era (before 1964) support the choice of testing models on measles data. Many other researchers formulated measles models and fit them to data, as in Bjørnstad et al. [29], where a time scale of two weeks is recommended fitting the number of cases, and in Yingcun Xia et al. [30], where a model is used to examine a spatial network. In this paper, we choose to use data of measles infections in the USA and UK in two decades of the pre-vaccination era (1944–1964) to compare the goodness of fit of ODEs and FDEs to those epidemics.

While ODEs are well-established as deterministic models of the spread of diseases [31,32], FDE models are sometimes used. However, often these approaches lack mathematical basis or physical interpretation except for exchanging integer differentiation with fractional ones [26,33]. Angstmann et al. [34] and Sardar et al. [35] provided a valid variation by considering the memory of the non-Markovian infection process. The result is a mixed system of integer and fractional derivatives of the Riemann-Liouville type. Saeedian et al. [36] showed how another memory functional of the process can lead to replacing the integer derivatives with Caputo fractional derivatives. In this paper, we show how Caputo fractional differential equations follow naturally from fractional stochastic processes like those introduced in literature [37,38,39,40,41,42,43,44,45,46]. We then compare transient and long term dynamics between the FDE and ODE models while fitting them to three different data sets. The Akaike Information Criterion (AIC) and Bayesian Information Criterion (BIC) are used to compare between the fits of the two models to three data sets. For completeness, we will cover all the required background and the relevant definitions in Section 2. That includes a synopsis of Caputo’s fractional calculus and fractional stochastic SEIR processes. Section 2 will also include the derivation of the fractional order differential equation depicting the SEIR model from the fractional stochastic SEIR process. It will be followed by the stability analysis of the equilibria of the system of fractional differential equations, which will be then fitted to measles data and simulated.

## 2. Methods

In this section, we provide a background for fractional differentiation and a fractional birth and death process. We also introduce the integer and fractional differential equations for the SEIR model and analyze the stability of the FDEs’ equilibria.

### 2.1. Preliminaries

#### Fractional Calculus

Let $D^{n}$ be the Leibniz integer-order differential operator given by

$$D^{n}f=\frac{d^{n}f}{dt^{n}}=f^{\left(n\right)},$$

and let $J^{n}$ be an integration operator of integer order given by

(1) $$J^{n}f\left(t\right)=\frac{1}{n!}\int_{0}^{t}{\left(t-\tau \right)}^{n-1}f\left(\tau \right)d\tau ,$$

where $n\in Z^{+}$. Let us use $D=D^{1}$ for the first derivative. We will use $\partial_{x}^{\alpha }F:=\frac{\partial^{\alpha }F}{\partial x^{\alpha }}$ and use $\partial_{x}F:=\frac{\partial F}{\partial x}$.

For fraction-order integrals, we use

(2) $$J^{n-\alpha }f\left(t\right)=\frac{1}{\Gamma (n-\alpha )}\int_{0}^{t}{\left(t-\tau \right)}^{n-\alpha -1}f\left(\tau \right)d\tau ,$$

where n−1<α≤n. Now, define the Caputo fractional differential operator $D_{*}^{\alpha }$ to be

$$D_{*}^{\alpha }f\left(t\right)=J^{n-\alpha }D^{n}f\left(t\right),$$

where n−1<α≤n, for n∈N. It is also known that

(3) $$\begin{array}{cc} \lim_{\alpha \to n}D_{*}^{\alpha }f\left(t\right) & =f^{\left(n\right)}\left(t\right),\\ \lim_{\alpha \to n-1}D_{*}^{\alpha }f\left(t\right) & =f^{\left(n-1\right)}\left(t\right)-f^{\left(n-1\right)}\left(0\right) \end{array}$$

for any n∈N. We will consider n=1 in this work; that is 0<α≤1. In that case,

(4) $$J^{1-\alpha }f\left(t\right)=\int_{0}^{t}f\left(\tau \right)dg_{t}\left(\tau \right),$$

where $g_{t}\left(\tau \right)=\frac{1}{\Gamma (2-\alpha )}\left(t^{1-\alpha }-{\left(t-\tau \right)}^{1-\alpha }\right)$. That is, for each *t*, the integral $J^{1-\alpha }f\left(t\right)$ is an area under f(τ), while above the curve of $g_{t}\left(\tau \right)$ that works as a deformed or slowed time-scale, as illustrated by Podlubny [47].

The generalized mean-value theorem for the Caputo fractional derivative is given as

$$f\left(x\right)=f\left(a\right)+\frac{1}{\Gamma (\alpha )}D_{*}^{\alpha }f\left(c\right){\left(x-a\right)}^{\alpha }\;\mathrm{for}\;\mathrm{some}\;\;a\leq c\leq x$$

and for all x∈(a,b] whenever $f,D_{*}^{\alpha }f\in C\left(\left[a,b\right]\right)$; see, e.g., Özalp and Demirci [48].

The Mittag-Leffler is a function that generalizes the exponential function. That function can be written as follows:

(5) $$E_{\alpha }\left(z\right)=\sum_{k=0}^{\infty }\frac{z^{k}}{\Gamma (\alpha k+1)},\;\alpha \in R^{+},\;z\in C,$$

or, more generally using two parameters,

(6) $$E_{\alpha ,\beta }\left(z\right)=\sum_{k=0}^{\infty }\frac{z^{k}}{\Gamma (\alpha k+\beta )},\;\alpha ,\beta \in R^{+},\;z\in C.$$

The general Mittag-Leffler has the following important property for any α,β>0:

(7) $$E_{\alpha ,\beta }\left(z\right)=zE_{\alpha ,\alpha +\beta }\left(z\right)+\frac{1}{\Gamma (\beta )}.$$

Two important differential properties of the Mittag-Leffler function are that

(8) $$D_{*}^{\alpha }e^{\lambda t}=t^{-\alpha }E_{1,1-\alpha }\left(\lambda t\right)$$

and

(9) $$D_{*}^{\alpha }E_{\alpha ,1}\left(\lambda t^{\alpha }\right)=\lambda E_{\alpha ,1}\left(\lambda t^{\alpha }\right)$$

for any λ>0.

### 2.2. Fractional Stochastic Process

**Figure 1:**
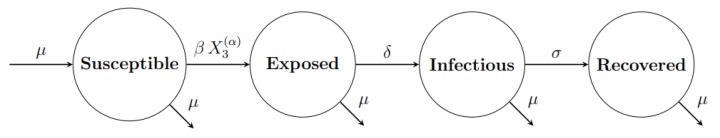
Schematic diagram of the susceptible-exposed-infectious-recovered (SEIR) model depicting transitions between different compartments.

Fix 0<α≤1. Following Earn et al. [49], we consider a compartmental susceptible-exposed- infected-recovered (SEIR) model to depict the measles transmission dynamics in a closed population. Let $X_{1}^{\left(\alpha \right)}$, $X_{2}^{\left(\alpha \right)}$, $X_{3}^{\left(\alpha \right)}$, and $X_{4}^{\left(\alpha \right)}$ be the number of susceptible, exposed, infected, and recovered individuals, respectively, such that $X_{1}^{\left(\alpha \right)}+X_{2}^{\left(\alpha \right)}+X_{3}^{\left(\alpha \right)}+X_{4}^{\left(\alpha \right)}=N$, the population size. Figure 1 shows how the disease is progressing from one sub-population to another.

Here, μ is the recruitment and per capita death rate, β is the transmission rate, δ is the rate at which exposed individuals become infectious, and σ is the recovery rate.

A stochastic SEIR model can be depicted using a continuous time Markov chain (CTMC) like the birth and death process with non-linear rates of transition as those given in Table 1; see Allen [50] (p. 22) and [51] (p. 321). Bartlett, M.S. [9] and Greenwood and Gordillo [31] introduced (integer) stochastic SIR model using CTMC with rates similar to those in the first six rows in Table 1 to show a deterministic SIR model of the ODE type depicting the approximate dynamics of the means of the processes. Here, we introduce a fractional SEIR model using a CTMC of fractional birth and death process on triplets (i,j,k) with rates provided by Table 1.

An α-fractional SEIR stochastic process $\left\{\left(X_{1}^{\left(\alpha \right)}\left(t\right),X_{2}^{\left(\alpha \right)}\left(t\right),X_{3}^{\left(\alpha \right)}\left(t\right)\right):t\geq 0\right\}$ for 0<α≤1 with state probabilities

$$p_{\left(i,j,k\right)}^{\left(\alpha \right)}\left(t\right)=P\left(\left(X_{1}^{\left(\alpha \right)}\left(t\right),X_{2}^{\left(\alpha \right)}\left(t\right),X_{3}^{\left(\alpha \right)}\left(t\right)\right)=\left(i,j,k\right)|\left(X_{1}^{\left(\alpha \right)}\left(0\right),X_{2}^{\left(\alpha \right)}\left(0\right),X_{3}^{\left(\alpha \right)}\left(0\right)\right)=\left(i_{0},j_{0},k_{0}\right)\right)$$

for i,j,k=0,1,…, such that 0≤i+j+k≤N and $P(\left(X_{1}^{\left(\alpha \right)}\left(0\right),X_{2}^{\left(\alpha \right)}\left(0\right),X_{3}^{\left(\alpha \right)}\left(0\right)\right)=\left(i_{0},j_{0},k_{0}\right))=1$, has a fractional forward Kolmogorov equation of the stochastic SEIR model similar to Equation (A1) and is given by

(10) $$\begin{array}{ll} D_{*}^{\alpha }p_{\left(i,j,k\right)}^{\left(\alpha \right)}\left(t\right) & =\mu Np_{\left(i-1,j,k\right)}^{\left(\alpha \right)}\left(t\right)+\beta \left(i+1\right)\frac{k}{N}p_{\left(i+1,j-1,k\right)}^{\left(\alpha \right)}\left(t\right)+\mu \left(i+1\right)p_{\left(i+1,j,k\right)}^{\left(\alpha \right)}\left(t\right)\\ & +\delta \left(j+1\right)p_{\left(i,j+1,k-1\right)}^{\left(\alpha \right)}\left(t\right)+\mu \left(j+1\right)p_{\left(i,j+1,k\right)}^{\left(\alpha \right)}\left(t\right)+\left(\sigma +\mu \right)\left(k+1\right)p_{\left(i,j,k+1\right)}^{\left(\alpha \right)}\left(t\right)\\ & \;-\left(\mu N+\beta i\frac{k}{N}+\mu i+\left(\delta +\mu \right)j+\left(\sigma +\mu \right)k\right)p_{\left(i,j,k\right)}^{\left(\alpha \right)}\left(t\right) \end{array},$$

with $p_{\left(i,j,k\right)}^{\left(\alpha \right)}\left(t\right)=0$ if either i,j, or *k* are negative or i+j+k>N (see Di Crescenzo et al. [45]). The classical forward Kolmogorov equation of the stochastic SEIR model follows when α=1 with state probabilities $p_{\left(i,j,k\right)}^{\left(1\right)}\left(t\right)$,^51^] (p. 321). Equation (10) can be used to find the probability generating function $G^{\left(\alpha \right)}\left(u,v,w,t\right)=E\left(u^{X_{1}^{\left(\alpha \right)}\left(t\right)}v^{X_{2}^{\left(\alpha \right)}\left(t\right)}w^{X_{3}^{\left(\alpha \right)}\left(t\right)}\right)$ of the state probabilities, as the solution of the Cauchy problem

(11) $$\begin{array}{ll} D_{*}^{\alpha }G^{\left(\alpha \right)} & =\mu N\left(u-1\right)G^{\left(\alpha \right)}+\mu \left(1-u\right)\partial_{u}G^{\left(\alpha \right)}+\left(\delta w+\mu -\left(\delta +\mu \right)v\right)\partial_{v}G^{\left(\alpha \right)}\\ & +\left(\sigma +\mu \right)\left(1-w\right)\partial_{w}G^{\left(\alpha \right)}+\beta \frac{w}{N}\left(v-u\right)\partial_{uw}G^{\left(\alpha \right)} \end{array}$$

for t>0 and $G^{\left(\alpha \right)}\left(u,v,w,0\right)=u^{i_{0}}v^{j_{0}}w^{k_{0}}$, for −1<u,v,w<1.

Note that the integer or classical stochastic SEIR process is $\left(X_{1}^{\left(1\right)}\left(t\right),X_{2}^{\left(1\right)}\left(t\right),X_{3}^{\left(1\right)}\left(t\right)\right)$ which is simply the case when α=1. Interestingly, the fractional stochastic SEIR process is a random-time subordination of the integer stochastic SEIR model, as established for other fractional processes like the fractional Poisson process [37,45,52], and the fractional birth and/or death processes [39,40,42,43,53]. In Mandelbrot and Taylor [54], the stochastic time process $T_{2\alpha }$ is called the operational time, and *t* is the physical time.

**Theorem** **1.** *The fractional stochastic SEIR process $\left(X_{1}^{\left(\alpha \right)}\left(t\right),X_{2}^{\left(\alpha \right)}\left(t\right),X_{3}^{\left(\alpha \right)}\left(t\right)\right)$ has the same distribution as the random-time subordinated integer stochastic SEIR process*

$$\left(X_{1}^{\left(1\right)}\left(T_{2\alpha }\left(t\right)\right),X_{2}^{\left(1\right)}\left(T_{2\alpha }\left(t\right)\right),X_{3}^{\left(1\right)}\left(T_{2\alpha }\left(t\right)\right)\right)$$

*for t>0 and 0<α≤1.*

The proof is provided in Appendix A.

### 2.3. Measles Model via Fractional Differential Equations (FDE)

The means of the three discrete-marginal processes $X_{1}^{\left(\alpha \right)}\left(t\right)$, $X_{2}^{\left(\alpha \right)}\left(t\right)$, and $X_{3}^{\left(\alpha \right)}\left(t\right)$ can be found using $\partial_{u}G^{\left(\alpha \right)}\left(1,1,1,t\right)$, $\partial_{v}G^{\left(\alpha \right)}\left(1,1,1,t\right)$, and $\partial_{w}G^{\left(\alpha \right)}\left(1,1,1,t\right)$, respectively. Let $S^{\left(\alpha \right)}\left(t\right):=\frac{1}{N}E\left(X_{1}^{\left(\alpha \right)}\left(t\right)\right)$, $E^{\left(\alpha \right)}\left(t\right):=\frac{1}{N}E\left(X_{2}^{\left(\alpha \right)}\left(t\right)\right)$, and $I^{\left(\alpha \right)}\left(t\right):=\frac{1}{N}E\left(X_{3}^{\left(\alpha \right)}\left(t\right)\right)$, where *N* is the total population size and E(x) is the expected value of *x*. Thus, using Equation (11) and approximating $E(X_{1}^{\left(\alpha \right)}\left(t\right)X_{3}^{\left(\alpha \right)}\left(t\right))$ by $E\left(X_{1}^{\left(\alpha \right)}\left(t\right)\right)E\left(X_{3}^{\left(\alpha \right)}\left(t\right)\right)$, we reach the fractional order version of the system of equations that was used by Bartlett, M.S. [9] to model measles:

(12) $$\begin{array}{cc} D_{*}^{\alpha }S^{\left(\alpha \right)} & =\mu -\beta S^{\left(\alpha \right)}I^{\left(\alpha \right)}-\mu S^{\left(\alpha \right)}\\ D_{*}^{\alpha }E^{\left(\alpha \right)} & =\beta S^{\left(\alpha \right)}I^{\left(\alpha \right)}-\left(\mu +\delta \right)E^{\left(\alpha \right)}\\ D_{*}^{\alpha }I^{\left(\alpha \right)} & =\delta E^{\left(\alpha \right)}-\left(\mu +\sigma \right)I^{\left(\alpha \right)} \end{array}$$

where $S^{\left(\alpha \right)}$, $E^{\left(\alpha \right)}$, and $I^{\left(\alpha \right)}$ be the proportion of susceptible, exposed, and infected individuals, respectively. With proportion of recovered individuals given by $R^{\left(\alpha \right)}=1-\left(S^{\left(\alpha \right)}+E^{\left(\alpha \right)}+I^{\left(\alpha \right)}\right)$, we reach the fractional α order SEIR model

(13) $$\begin{array}{cc} D_{*}^{\alpha }S^{\left(\alpha \right)} & =\mu -\beta S^{\left(\alpha \right)}I^{\left(\alpha \right)}-\mu S^{\left(\alpha \right)}\\ D_{*}^{\alpha }E^{\left(\alpha \right)} & =\beta S^{\left(\alpha \right)}I^{\left(\alpha \right)}-\left(\mu +\delta \right)E^{\left(\alpha \right)}\\ D_{*}^{\alpha }I^{\left(\alpha \right)} & =\delta E^{\left(\alpha \right)}-\left(\mu +\sigma \right)I^{\left(\alpha \right)}\\ D_{*}^{\alpha }R^{\left(\alpha \right)} & =\sigma I^{\left(\alpha \right)}-\mu R^{\left(\alpha \right)} \end{array}$$

with 0<α≤1. The non-negative parameters β, μ, δ, and σ—denoting them by θ, for brevity—have dimensions given by $\frac{1}{{\mathrm{time}}^{\alpha }}$. By construction of the FDE model as a mean field approximation of the α-fractional stochastic SEIR process which, in its turn, is a subordination of an integer stochastic SEIR process by Theorem 1, those parameters could be interpreted as the rates measured by an independent observer of the process or calculated based on a cosmic time flow [47]. We replace those parameters with a power α of new parameters; that is, $\theta_{*}^{\alpha }$ in place of θ so the parameters $\theta_{*}$ will have the dimension of $\frac{1}{time}$, and the system becomes the following form:

(14) $$\begin{array}{cc} D_{*}^{\alpha }S^{\left(\alpha \right)} & =\mu_{*}^{\alpha }-\beta_{*}^{\alpha }S^{\left(\alpha \right)}I^{\left(\alpha \right)}-\mu_{*}^{\alpha }S^{\left(\alpha \right)}\\ D_{*}^{\alpha }E^{\left(\alpha \right)} & =\beta_{*}^{\alpha }S^{\left(\alpha \right)}I^{\left(\alpha \right)}-\left(\mu_{*}^{\alpha }+\delta_{*}^{\alpha }\right)E^{\left(\alpha \right)}\\ D_{*}^{\alpha }I^{\left(\alpha \right)} & =\delta_{*}^{\alpha }E^{\left(\alpha \right)}-\left(\mu_{*}^{\alpha }+\sigma_{*}^{\alpha }\right)I^{\left(\alpha \right)}\\ D_{*}^{\alpha }R^{\left(\alpha \right)} & =\sigma_{*}^{\alpha }I^{\left(\alpha \right)}-\mu_{*}^{\alpha }R^{\left(\alpha \right)}. \end{array}$$

By replacing θ by $\theta_{*}^{\alpha }$, the system of integer order differential equations with its epidemiological parameters follows directly from the system of fractional order differential equations when α=1.

### 2.4. Measles Model via Ordinary Differential Equations (ODE)

The following system of differential equations represents the ordinary differential equation representation of the SEIR model and is the FDE model when α=1 in Equation (14).

(15) $$\begin{array}{cc} DS & =\mu -\beta SI-\mu S\\ DE & =\beta SI-(\mu +\delta )E\\ DI & =\delta E-(\mu +\sigma )I\\ DR & =\sigma I-\mu R \end{array},$$

where μ,β,δ, and σ are the model parameters described above. They all have dimensions given by $\frac{1}{\mathrm{time}}$. The last Equation in (15) is redundant since R=1−(S+E+I).

### 2.5. Measles Model via α-Dependent Ordinary Differential Equations

We are interested in comparing the FDE versus ODE modeling approaches. It is important to note that the basic ODE case considers α=1; however, in the FDE case, α appears in the derivative, as well as the parameter, values. In order to better compare these two approaches, here, we develop an ODE analogue to the FDE that incorporates α in the parameter values. We call this new system the α-dependent ODE. By dropping the α order derivative from the left side and α power from $S^{\left(\alpha \right)}$, $E^{\left(\alpha \right)}$, and $I^{\left(\alpha \right)}$ of Equation (14), our α-dependent ODE takes the following form:

(16) $$\begin{array}{cc} DS & =\mu_{*}^{\alpha }-\beta_{*}^{\alpha }SI-\mu_{*}^{\alpha }S\\ DE & =\beta_{*}^{\alpha }SI-\left(\mu_{*}^{\alpha }+\delta_{*}^{\alpha }\right)E\\ DI & =\delta_{*}^{\alpha }E-\left(\mu_{*}^{\alpha }+\sigma_{*}^{\alpha }\right)I\\ DR & =\sigma_{*}^{\alpha }I-\mu_{*}^{\alpha }R. \end{array}$$

Here, the above systems may differ from the classical ODE (Equation (15)) if α≠1. In this case, the α value used in the above α-dependent ODE are obtained from data fitting procedures using the FDE model (Equation (14)).

### 2.6. Model Analysis

Analysis of the ODE is almost the same as of the FDE, so we include the FDE one here. We start by proving the positive invariance of the region of solutions of the FDE model. Henceforth, we drop the α from $S^{\left(\alpha \right)}$, $E^{\left(\alpha \right)}$, and $I^{\left(\alpha \right)}$, for brevity.

The following two lemmas of asymptotic behavior of FDEs are given here and their proof in Appendix A for completeness.

**Lemma** **1.** *The closed simplex region $M=\{\left(S,E,I\right)\in R_{+}^{3}:0\leq S+E+I\leq 1\}$ is a positive invariant set for the FDE model in Equation (14).*

We can find the model’s equilibrium points by setting $D_{*}^{\alpha }S=0$, $D_{*}^{\alpha }E=0$, and $D_{*}^{\alpha }I=0$. Thus, there are two equilibria to the measles SEIR model (14). They are:

1. the disease free equilibrium DFE≡(1,0,0) and
2. the endemic equilibrium

(17) $$EE=\left(s^{*},e^{*},i^{*}\right)\equiv \left(\frac{1}{R_{0}},\frac{\mu }{\delta +\mu }\left(1-\frac{1}{R_{0}}\right),\frac{\mu }{\beta }\left(R_{0}-1\right)\right),$$

where the basic reproduction number is $R_{0}:=\frac{\beta \delta }{\left(\mu +\sigma )(\mu +\delta \right)}$. EE exists only when $1<R_{0}<1+\frac{\beta }{\mu }$.

**Remark** **1.** *An equilibrium is locally asymptotically stable if the eigenvalues of the Jacobian matrix of the n-dimensional system, namely $\lambda_{1},\lambda_{2},\ldots ,\lambda_{n}$, have the property that $|\arg\left(\lambda_{i}\right)|>\frac{\alpha \pi }{2}$, for i=1,2,…,n,* [25] (p. 158).

Thus, in general, the stability of the ordinary differential equations model implies stability of its fractional counter model of order α. But, here, they are equivalent due to the following lemma, in which the solution could be found in Appendix A.

**Lemma** **2.** *The Disease-Free Equilibrium DFE is locally asymptotically stable if $R_{0}<1$. The endemic equilibrium EE is is locally asymptotically stable if $R_{0}>1$.*

Therefore, they have the same asymptotic behavior. Yet, the transient behavior differs, as will be seen by simulations below.

Moreover, a very important difference is their oscillation behavior is not similar. Let $\lambda_{\ell }$ and $u_{\ell }$ for ℓ=1,2,…,n be the eigenvalues and their respective eigenvectors of an n×n matrix *A*. The general solution of initial value problem consisting of a system of *n* linear fractional differential equations $D_{*}^{\alpha }x\left(t\right)=Ax\left(t\right)$, such that $x\left(0\right)=x_{0}$, can be found to be

(18) $$x\left(t\right)=\sum_{\ell =1}^{n}c_{\ell }u_{\ell }E_{\alpha }\left(\lambda_{\ell }t^{\alpha }\right)$$

for certain constants $c_{\ell }\in C$ for ℓ=1,2,…,n, such that $\sum_{\ell =1}^{n}c_{\ell }u_{\ell }=x_{0}$,^25^] (Theorem 7.13). In case that α=1, we recover the known solution of the system of ODEs given by

$$x\left(t\right)=\sum_{\ell =1}^{n}c_{\ell }u_{\ell }\exp\left(\lambda_{\ell }t\right).$$

If n=3 and *A* is not a symmetric matrix, then at least one of the eigenvalues is a real-valued number and the other two eigenvalues, say $\lambda_{2}$ and $\lambda_{3}$, are conjugate complex-valued. In that situation, x(t) would oscillate with inter-peak periods, called inter-epidemic period in disease modeling, given by $2\pi {\left(Im\left(\lambda_{2}\right)\right)}^{-1}$ [14]. If $Re(\lambda_{\ell })<0$ for all *ℓ*, then the oscillations will be damped to zero. That damped oscillation is straightforward in the case of α=1 due to the exponential damping in the superposition of the sine and cosine functions. That behavior, however, is not straightforward for 0<α<1.

### 2.7. Numerical Simulations

Since the mean of the subordinator process is $E\left(T_{\alpha }\left(t\right)\right)=\frac{t^{\alpha }}{\Gamma (\alpha +1)}$, we use a method similar to that which was introduced in Demirici and Özalp [55] to find approximate solutions to initial value FDE problems. We use that method here to simulate the solution of the FDE measles SEIR model. Consider the initial value problem:

(19) $$\begin{array}{cc} D_{*}^{\alpha }x\left(t\right) & =f(t,x(t)),\;\mathrm{for}\;0<t\leq T,\\ x(0) & =x_{0}, \end{array}$$

for some T>0. A solution of Equation (19) is approximated by the deterministic time subordination

(20) $$x\left(t\right)=y\left(\frac{t^{\alpha }}{\Gamma (\alpha +1)}\right),$$

of y(s), the solution of the ordinary differential equation

(21) $$\begin{array}{cc} \frac{dy(s)}{ds} & =g\left(s,y\left(s\right)\right),\;\mathrm{for}\;0<s\leq \frac{t^{\alpha }}{\Gamma (\alpha +1)}\\ y(0) & =x_{0}. \end{array},$$

where

(22) $$\begin{array}{c} g\left(s,y\left(s\right)\right)=f(t-{\left(t^{\alpha }-s\Gamma \left(\alpha +1\right)\right)}^{\frac{1}{\alpha }},x\left(t-{\left(t^{\alpha }-s\Gamma \left(\alpha +1\right)\right)}^{\frac{1}{\alpha }}\right)) \end{array}$$

for all 0<t≤T,^55^].

We use the subordination of the solution of ODEs to FDEs represented in Equations (20) and (21) to numerically simulate solutions of FDEs; see Algorithm 1.
**Algorithm 1** Numerical solution of $D_{*}^{\alpha }x\left(t\right)=f\left(t,x\left(t\right)\right)$ for 0<t<T with $x\left(0\right)=x_{0}$.Input: α,T,f(t,x(t)),m,n Output: x(t)**begin** Divide the interval [0,T] into *n* sub-intervals using
$$0=t_{0}<t_{1}<\ldots <t_{n}=T.$$**for**i=1,2,…,n Divide the interval $\left[0,\frac{t_{i}^{\alpha }}{\Gamma (\alpha +1)}\right]$ into further *m* sub-intervals using
$$0=s_{0}<s_{1}<\ldots <s_{m}=\frac{t_{i}^{\alpha }}{\Gamma (\alpha +1)}.$$ Solve the system $Dy\left(s\right)=f(t_{i}-{\left(t_{i}^{\alpha }-s\Gamma \left(\alpha +1\right)\right)}^{\frac{1}{\alpha }},y\left(s\right))$ with $y\left(0\right)=x_{0}$ using Euler or Runge-Kutta methods on $s_{0},s_{1},\ldots ,s_{m}$. Retain $x\left(t_{i}\right)=y\left(s_{m}\right)$.**end** Return $\left[x_{0},x\left(t_{1}\right),x\left(t_{2}\right),\ldots ,x\left(t_{n}\right)\right]$.**end**

### 2.8. Fitting FDE and ODE Models to Measles Data

We use the method of ordinary least squares (OLS) to fit the FDE model to the data by minimizing the objective function

$$L\left(\alpha ,\beta ,\mu ,\delta ,\sigma \right)=\sum_{i=1}^{n}{\left(I_{i}-{\hat{I}}_{i}\right)}^{2}$$

for α∈(0,1], and β,μ,δ,σ∈(0,∞), where $\left\{I_{i},i=1,\ldots ,n\right\}$ is the data of actual proportion of infections and $\left\{{\hat{I}}_{i},i=1,\ldots ,n\right\}$ is the simulated proportion of infections. The values ${\hat{I}}_{i}$ approximating $I(t_{i})$ are found by solving the FDE model using Algorithm 1.

Parameter estimation was conducted using MATLAB MultiStart and fmincon functions. The MultiStart function carries out the optimization procedure using initial points within the parameters’ spaces. It generates some initial points depending on a converging algorithm. The fmincon function finds a local minimum for the constrained nonlinear multivariable function. The MultiStart, together with fmincon functions, does the global optimization of a nonlinear multivariable function. The MultiStart function uses parallel processing, which drastically reduces the running time.

## 3. Results

We solve the system of FDE (Equation (14)) using Algorithm 1 and the systems of ODE (Equations (15) and (16)) using the Runge-Kutta method.

**Figure 2:**
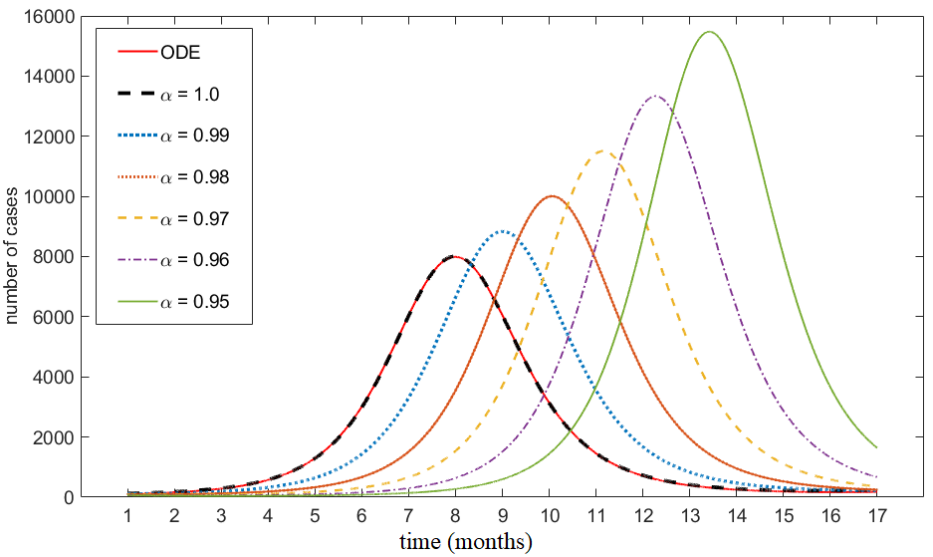
Number of cases using classical ordinary differential equation (ODE) model and fractional differential equation (FDE) model with different fractional orders α. The simulations are done using $\mu =\mu_{⋆}=0.0027,$ $\beta =\beta_{⋆}=119.2257,$ $\delta =\delta_{⋆}=16.7301,\;\sigma =\sigma_{⋆}=10.1873,$ and N=7781984.

**Figure 3:**
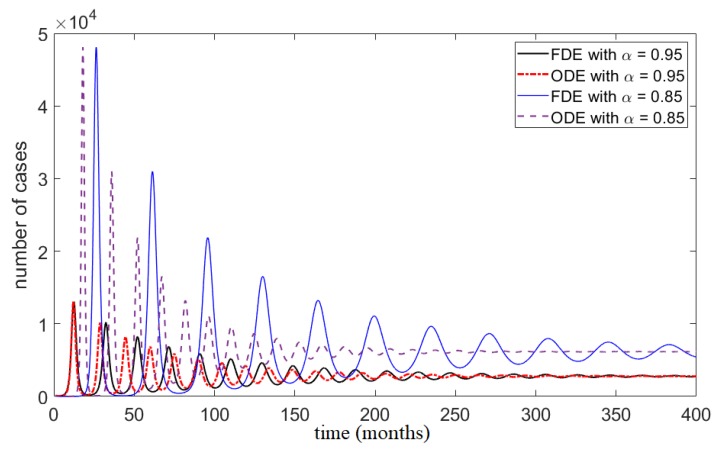
Number of cases using FDE model and its analogous α-dependent ODE with different fractional orders α. The simulations are done using $\mu_{⋆}=0.0027,\;\beta_{⋆}=119.2257,\;\delta_{⋆}=16.7301,$ and $\sigma_{⋆}=10.1873$.

In order to study the qualitative aspects of the FDE and its numerical solution, we carried out a number of simulations. Simulations of the classical ODE (Equation (15)) and FDE (Equation (14)), Figure 2, shows that the system of fractional differential equations is very sensitive to its order of differentiation α. For smaller α, the peak number of cases of the epidemic is larger but the duration of the outbreak is shorter. The solution of the FDE model converges to the solution of the classical ODE as α→1. To further compare the two modeling approaches, we consider the analogue ODEs derived for specific α values; see Equation (16). These comparisons are shown in Figure 3. During transient dynamics both models exhibit several peaks in the number of cases. The number of these peaks and their respective amplitudes are similar between models; however, there are differences in the timing of these peaks. The transient oscillations of the FDE model are more stretched out than its ODE analogue, and its solutions experience longer inter-epidemic times. Both models approach the same equilibria solutions.

**Figure 4:**
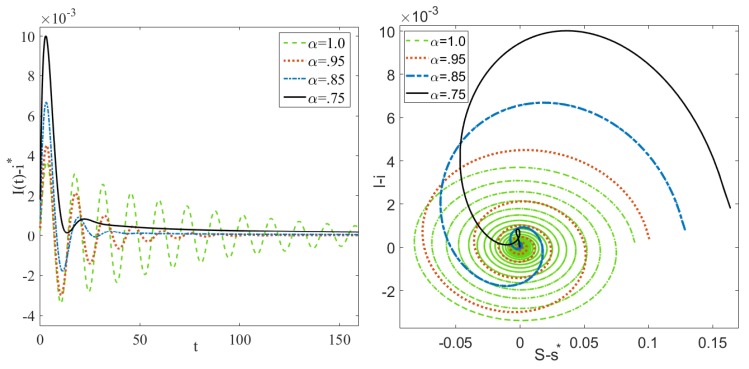
Simulations of solutions of the SEIR FDE centered about the endemic equilibrium (EE) for α = 1, 0.95, 0.85, and 0.75 using Equation (18) shows a suppression of damped oscillations as α decreases. The simulations are done using $\mu_{⋆}=0.0027,\;\beta_{⋆}=119.2257,\delta_{⋆}=16.7301,$ and $\sigma_{⋆}=10.1873$.

Simulations of Equation (18), Figure 4, shows that disease models of fractional order equations lack the same oscillatory behavior exhibited by systems of ODEs with conjugate complex eigenvalues of the Jacobian matrices calculated at endemic equilibrium.

**Figure 5:**
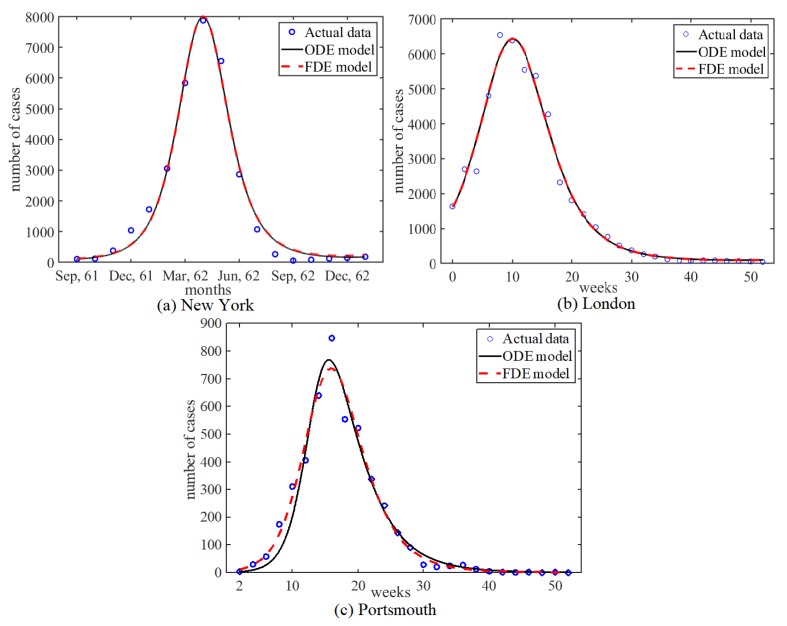
Simulations of ODE and FDE fitted to measles epidemics in the pre-vaccination era for (**a**) New York, (**b**) London, and (**c**) Portsmouth using the parameter values from Table A4. ODE and FDE models give similar fitting in (**a**) and (**b**) but FDE model performs better than ODE model in (**c**).

The models were fitted to three measles epidemics in the pre-vaccination era in three different cities: New York, London, and Portsmouth. Simulations of the fitted ODE and FDE models are shown in Figure 5. See Appendix B for the data and the parameter estimates, as well.

The estimates of α are 0.999999999524,0.999999999614, and 0.875784505546 for New York, London, and Portsmouth, respectively. The values obtained for α for the New York and London data sets are very close to one, where the FDE model converges to the ODE model (Equation (15)). Statistical measurements on the data fittings are presented in Table A4, found in Appendix B.4. The Akaike information criterion (AIC), Bayesian information criterion (BIC), and Sum squared error (SSE) are very similar between the two models for all three data sets. However, the biggest differences between the two models are found with the Portsmouth data fittings where the FDE performs slightly better than the ODE.

## 4. Discussion

Replacing first order derivatives with Caputo fractional derivatives has been the practice for many studies using fractional order modeling of diseases. In this paper, we show how these models follow from an approximation to the dynamical system governing the means of fractional stochastic SEIR processes. Moreover, we study ordinary and fractional order systems of differential equations of SEIR models using three data sets of measles epidemics in three different cities selected from the pre-vaccination era. Two of the data sets fits resulted in α values of extremely close to one, with differences appearing in the ninth decimal digit. In these two cases, the two models are equivalent, apart from round off errors potentially from the numerical methods. We suspect these round off error are also causing the extremely slight differences in the statistical measures (AIC, BIC, SSE) of the data fittings. In the third case, the data for Portsmouth, where α=0.88 the FDE model outperformed the ODE model; see Figure 5 and Appendix B.4.

Angstmann et al. [34] use the master equation of a continuous-time random walk to derive an FDEM involving Riemann-Liouville fractional derivatives. Power laws are postulated to model time of infectiousness and recovery. That extension from exponential times in ordinary differential equations is a different approach from the mean field approximation of a stochastic process. Saeedian et al. [36] introduced the Caputo fractional differential equations through a memory of the whole process of infection and disease recovery. In our paper, we have considered, for the first time, fractional stochastic SEIR model and have shown how the Caputo fractional differential equations follows as mean-field approximation of the process.

Fractional stochastic SEIR model introduced here turns out to be a random-time subordination of a classical stochastic SEIR model. Other real-life systems are modeled using a subordination of a stochastic process. A subordinated process was introduced by Mandelbrot and Taylor [54] to model the logarithm of market prices where the original process is a Brownian motion subordinated by a stochastic time process $T_{2\alpha }$, which is the same random time process we have found here.

Further study of the fractional stochastic SEIR model might lead to interesting dynamical behaviors. For instance, it can provide more insights into the stochastic oscillations of the disease in a more flexible way than their classical counterparts. Thus, studying the fractional stochastic SEIR model is the next step in this work.

## 5. Conclusions

In this paper, we compare two deterministic models of disease: ordinary differential equations (ODE) and fractional differential equations (FDE). We use three different data sets of measles epidemics from the pre-vaccination era. We also explain FDEs as the mean-field approximation of a fractional stochastic SEIR model. To our knowledge, this is the first time such a fractional stochastic process is introduced and connected to the fractional order differential equations.

We conclude that, depending on the specific data set, the FDE model either converges to the ODE model and fits data similarly, or fits the data better and outperforms the ODE model. It is worth asking whether the complexities introduced by FDE approach are worth pursuing, as the numerical algorithms for solving FDEs are computationally expensive. Here, the FDE approach yielded better results in only one of three data sets, and the results were still similar to those obtained by the ODE. However, other data sets may yield larger differences between these two modeling approaches and benefit more from an FDE approach. In particular, more refined and larger data sets may be more appropriate for this approach. Here, oscillations can persist longer in the FDE solutions than those of the ODE (Figure 3, α=0.85), suggesting perhaps refined data set with seasonal variations may be good candidates for the FDE modeling approach. It is also important to note that the numerical methods for solving FDEs and data fitting procedures can be improved. Future studies, with good empirical data sets, should consider the FDE approach to modeling epidemics, as well as improving numerical algorithms.

## Figures and Tables

**Table 1:** Transitions and their rates for a birth and death process depicting a stochastic SEIR model.

Transition	Rate
$X_{1}^{\left(\alpha \right)}\to X_{1}^{\left(\alpha \right)}+1$	μN
$X_{1}^{\left(\alpha \right)}\to X_{1}^{\left(\alpha \right)}-1$	$\beta X_{1}^{\left(\alpha \right)}\frac{X_{3}^{\left(\alpha \right)}}{N}+\mu X_{1}^{\left(\alpha \right)}$
$X_{2}^{\left(\alpha \right)}\to X_{2}^{\left(\alpha \right)}+1$	$\beta X_{1}^{\left(\alpha \right)}\frac{X_{3}^{\left(\alpha \right)}}{N}$
$X_{2}^{\left(\alpha \right)}\to X_{2}^{\left(\alpha \right)}-1$	$\left(\mu +\delta \right)X_{2}^{\left(\alpha \right)}$
$X_{3}^{\left(\alpha \right)}\to X_{3}^{\left(\alpha \right)}+1$	$\delta X_{2}^{\left(\alpha \right)}$
$X_{3}^{\left(\alpha \right)}\to X_{3}^{\left(\alpha \right)}-1$	$\left(\mu +\sigma \right)X_{3}^{\left(\alpha \right)}$
$X_{4}^{\left(\alpha \right)}\to X_{4}^{\left(\alpha \right)}+1$	$\sigma X_{3}^{\left(\alpha \right)}$
$X_{4}^{\left(\alpha \right)}\to X_{4}^{\left(\alpha \right)}-1$	$\mu X_{4}^{\left(\alpha \right)}$

## Appendix A. Some Definitions and Proofs

Laplace transform of a function f(t) is defined as

$$L\left(f\right)\left(s\right)=\hat{f}\left(s\right)=\int_{0}^{\infty }e^{-st}f\left(t\right)dt.$$

The inverse transform is defined by

$$L^{-1}\left(\hat{f}\right)\left(t\right)=\frac{1}{2\pi i}\int_{C}e^{st}\hat{f}\left(s\right)ds$$

where C is a contour parallel to the imaginary axis and to the right of the singularities of $\hat{f}$. The Laplace transform of the Caputo fractional derivative is given by

$$L\left(D_{*}^{\alpha }f\right)\left(s\right)=s^{\alpha }\hat{f}\left(s\right)-s^{\alpha -1}f\left(0\right).$$

### Fractional Birth and Death Process

An α-fractional nonlinear birth and death process $\left\{N_{\alpha }\left(t\right):t\geq 0\right\}$ for 0<α≤1 with state probabilities

$$p_{n}^{\alpha }\left(t\right)=P\left(N_{\alpha }\left(t\right)=n|N_{\alpha }\left(0\right)=1\right)$$

for n≥0 is defined through the forward Kolmogorov (difference-)differential equations

(A1) $$D_{*}^{\alpha }p_{n}^{\alpha }\left(t\right)=\lambda_{n-1}p_{n-1}^{\alpha }\left(t\right)+\mu_{n+1}p_{n+1}^{\alpha }\left(t\right)-\left(\lambda_{n}+\mu_{n}\right)p_{n}^{\alpha }\left(t\right)$$

for n≥0 [39,43,53]. The rates $\lambda_{n}$ and $\mu_{n}$ are non-negative. The classical birth and death process follows when α=1 with state probabilities $p_{n}^{1}\left(t\right)$. When $\lambda_{n}=\lambda$ and $\mu_{n}=0$ for all *n*, the α-fractional nonlinear birth and death process becomes the α-fractional Poisson process [37,41,46]. There, it has shown that $N_{\alpha }\left(t\right)$ has the same probability distribution as $N(T_{\alpha }\left(t\right))$, where N(t) is the classical birth and death process which is independent of a random time process $T_{\alpha }\left(t\right)$; that is, a birth and death process subordinated by an α-stable time process.

The random time process $T_{\alpha }\left(t\right)$ has a distribution given by the folded solution of the fractional diffusion equation $\partial_{t}^{\alpha }F=\partial_{x}^{2}F$ for 0<α≤2, x∈R, t>0, and subject to F(x,0)=δ(x) for 0<α≤2 and $\partial_{t}^{\alpha }F\left(x,0\right)=0$ for 1<α≤2,^43^]. We will denote its measure by $\nu_{\alpha ,t}\left(ds\right):=P\left(T_{\alpha }\left(t\right)\in ds\right)$. It has a Laplace transform

$$L\left(\nu_{\alpha ,\cdot }\left(ds\right)\right)\left(r\right)=\int_{0}^{\infty }e^{-rt}\nu_{\alpha ,t}\left(ds\right)dt=r^{\frac{\alpha }{2}-1}e^{-sr^{\frac{\alpha }{2}}}ds$$

and moments $E\left[{\left(T_{\alpha }\left(t\right)\right)}^{k}\right]=\Gamma \left(k+1\right)\frac{t^{k\alpha }}{\Gamma (k\alpha +1)}$ for k=1,2,…; [46,56].

Note that, the absolute values of partial derivatives of *G* are finite; that is, $|\partial_{u,v,w}^{\left(i,j,k\right)}G|<\infty$ for any i,j,k=0,1,2,…. That is true since |u|,|v|,|w|<1 and the population size is finite. Thus, switching integrals with derivatives or summations below are valid.

**Proof of the Theorem** **1.** We are going to show that Laplace transform of the probability generating function of the process

$$\left(X_{1}^{\left(1\right)}\left(T_{2\alpha }\left(t\right)\right),X_{2}^{\left(1\right)}\left(T_{2\alpha }\left(t\right)\right),X_{3}^{\left(1\right)}\left(T_{2\alpha }\left(t\right)\right)\right)$$

is the same as Laplace transform $\hat{G}$ of *G*, that solves Equation (11). From there we will conclude that the two probability distributions are the same since the probability generating function of $\left(X_{1}^{\left(\alpha \right)}\left(t\right),X_{2}^{\left(\alpha \right)}\left(t\right),X_{3}^{\left(\alpha \right)}\left(t\right)\right)$, by construction, is also a solution to the Cauchy problem in Equation (11). From Equation (11), the Laplace transform $\hat{G}$ is the solution of

(A2) $$\begin{array}{ll} s^{\alpha }{\hat{G}}^{\left(\alpha \right)}-s^{\alpha -1}u^{i_{0}}v^{j_{0}}w^{k_{0}} & =\mu N\left(u-1\right){\hat{G}}^{\left(\alpha \right)}+\mu \left(1-u\right)\partial_{u}{\hat{G}}^{\left(\alpha \right)}+\left(\delta w+\mu -\left(\delta +\mu \right)v\right)\partial_{v}{\hat{G}}^{\left(\alpha \right)}\\ & +\left(\sigma +\mu \right)\left(1-w\right)\partial_{w}{\hat{G}}^{\left(\alpha \right)}+\beta \frac{w}{N}\left(v-u\right)\partial_{uw}{\hat{G}}^{\left(\alpha \right)} \end{array}$$

Let $H^{\left(\alpha \right)}\left(u,v,w,t\right)$ be the probability generating function of the state probabilities

$$q_{\left(i,j,k\right)}^{\left(\alpha \right)}\left(t\right)=P\left(\left(X_{1}^{\left(1\right)}\left(T_{2\alpha }\left(t\right)\right),X_{2}^{\left(1\right)}\left(T_{2\alpha }\left(t\right)\right),X_{3}^{\left(1\right)}\left(T_{2\alpha }\left(t\right)\right)\right)=\left(i,j,k\right)\right|$$

$$\left(X_{1}^{\left(1\right)}\left(T_{2\alpha }\left(0\right)\right),X_{2}^{\left(1\right)}\left(T_{2\alpha }\left(0\right)\right),X_{3}^{\left(1\right)}\left(T_{2\alpha }\left(0\right)\right)\right)=\left(i_{0},j_{0},k_{0}\right)).$$

That means that

$$\begin{array}{ll} H^{\left(\alpha \right)}\left(u,v,w,t\right) & =E\left(u^{X_{1}^{\left(1\right)}\left(T_{2\alpha }\left(t\right)\right)}v^{X_{2}^{\left(1\right)}\left(T_{2\alpha }\left(t\right)\right)}w^{X_{3}^{\left(1\right)}\left(T_{2\alpha }\left(t\right)\right)}\right)\\ & =\sum_{i}\sum_{j}\sum_{k}u^{i}v^{j}w^{k}q_{\left(i,j,k\right)}^{\left(\alpha \right)}\left(t\right)\\ & =\sum_{i}\sum_{j}\sum_{k}u^{i}v^{j}w^{k}\int_{0}^{\infty }p_{\left(i,j,k\right)}^{\left(1\right)}\left(s\right)\nu_{2\alpha ,t}\left(ds\right)\\ & =\int_{0}^{\infty }\left(\sum_{i}\sum_{j}\sum_{k}u^{i}v^{j}w^{k}p_{\left(i,j,k\right)}^{\left(1\right)}\left(s\right)\right)\nu_{2\alpha ,t}\left(ds\right)\\ & =\int_{0}^{\infty }G^{\left(1\right)}\left(u,v,w,s\right)\nu_{2\alpha ,t}\left(ds\right). \end{array}$$

Thus, the Laplace transform of the probability generating function $H^{\left(\alpha \right)}$ is given by

$$\begin{array}{ll} {\hat{H}}^{\left(\alpha \right)}\left(u,v,w,r\right) & =\int_{0}^{\infty }e^{-rt}\int_{0}^{\infty }G^{\left(1\right)}\left(u,v,w,s\right)\nu_{2\alpha ,t}\left(ds\right)dt\\ & =r^{\alpha -1}\int_{0}^{\infty }G^{\left(1\right)}\left(u,v,w,s\right)e^{-sr^{\alpha }}ds\\ & =r^{\alpha -1}{\hat{G}}^{\left(1\right)}\left(u,v,w,r^{\alpha }\right) \end{array}$$

Now, the Laplace transform of the probability generating function of the process $\left(X_{1}^{\left(1\right)}\left(t\right),X_{2}^{\left(1\right)}\left(t\right),X_{3}^{\left(1\right)}\left(t\right)\right)$ also solves (A2) when α=1 which is

(A3) $$\begin{array}{ll} s{\hat{G}}^{\left(1\right)}-u^{i_{0}}v^{j_{0}}w^{k_{0}} & =\mu N\left(u-1\right){\hat{G}}^{\left(1\right)}+\mu \left(1-u\right)\partial_{u}{\hat{G}}^{\left(1\right)}+\left(\delta w+\mu -\left(\delta +\mu \right)v\right)\partial_{v}{\hat{G}}^{\left(1\right)}\\ & +\left(\sigma +\mu \right)\left(1-w\right)\partial_{w}{\hat{G}}^{\left(1\right)}+\beta \frac{w}{N}\left(v-u\right)\partial_{uw}{\hat{G}}^{\left(1\right)}. \end{array}$$

If we substitute with $s=r^{\alpha }$ in Equation (A3) and multiply both sides by $r^{\alpha -1}$ we get

(A4) $$\begin{array}{ll} r^{\alpha }{\hat{H}}^{\left(\alpha \right)}-r^{\alpha -1}u^{i_{0}}v^{j_{0}}w^{k_{0}} & =\mu N\left(u-1\right){\hat{H}}^{\left(\alpha \right)}+\mu \left(1-u\right)\partial_{u}{\hat{H}}^{\left(\alpha \right)}+\left(\delta w+\mu -\left(\delta +\mu \right)v\right)\partial_{v}{\hat{H}}^{\left(\alpha \right)}\\ & +\left(\sigma +\mu \right)\left(1-w\right)\partial_{w}{\hat{H}}^{\left(\alpha \right)}+\beta \frac{w}{N}\left(v-u\right)\partial_{uw}{\hat{H}}^{\left(\alpha \right)} \end{array}$$

which is the same as Equation (A2). This completes the proof. □

**Proof of Lemma** **1.** Note that the Mittag-Leffler function $E_{\alpha ,1}\left(-\mu t^{\alpha }\right)\in \left(0,1\right)$ for t>0; see, e.g., Reference [46]. Starting on the S-axis when E(0)=I(0)=0 and $1\geq S\left(0\right)=S_{0}\geq 0$, then

$$S\left(t\right)=\frac{1}{\mu }+\left(S_{0}-\frac{1}{\mu }\right)E_{\alpha ,1}\left(-\mu t^{\alpha }\right)\geq 0$$

since μ>0 and t≥0. Starting on the E-axis when S(0), I(0)=0 and $E\left(0\right)=E_{0}\geq 0$, then

$$E\left(t\right)=E_{\alpha ,1}\left(-\left(\mu +\delta \right)t^{\alpha }\right)E_{0}\geq 0$$

Starting on the I-axis when S(0),E(0)=0 and $I\left(0\right)=I_{0}\geq 0$, then

$$\left(t\right)=E_{\alpha ,1}\left(-\left(\mu +\sigma \right)t^{\alpha }\right)I_{0}\geq 0$$

Thus, all axes are positive invariant, for S(0),E(0),I(0)≥0. If the solution of the system is leaving through the positive quadrant of the E-I plane, then $S(t_{e})$ = 0, and $E(t_{e})$ and $I(t_{e})>0$ for some $t_{e}>0$, such that $S\left(t\right)\leq S\left(t_{e}\right)$, for all $t>t_{e}$. But, $D_{*}^{\alpha }{S|}_{t=t_{e}}=\mu >0$. By the generalized mean value theorem

$$S\left(t\right)=S\left(t_{e}\right)+\frac{1}{\Gamma (\alpha )}D_{*}^{\alpha }S\left(\tau \right){\left(t-t_{e}\right)}^{\alpha }$$

for some $t_{e}\leq \tau <t$, then $S\left(t\right)>S\left(t_{e}\right)$ contradicting the original statement. The same argument could be used for the positive quadrant of the S-I plane with $D_{*}^{\alpha }{E|}_{t=t_{e}}=\beta S\left(t_{e}\right)I\left(t_{e}\right)>0$ and for the positive quadrant of the E-S plane with $D_{*}^{\alpha }{I|}_{t=t_{e}}=\alpha E\left(t_{e}\right)>0$. To show that S(t)+E(t)+I(t)≤1 for all t>0, if S(0)+E(0)+I(0)≤1,

(A5) $$\begin{array}{cc} D_{*}^{\alpha }\left(S+E+I\right) & =\mu -\mu (S+E+I)-\sigma I\\ \; & \leq \mu -\mu (S+E+I) \end{array}$$

Thus,

(A6) $$\begin{array}{cc} S(t)+E(t)+I(t) & \leq t^{\alpha }E_{\alpha ,\alpha +1}\left(-\mu t^{\alpha }\right)\mu +E_{\alpha ,1}\left(-\mu t^{\alpha }\right)\left(S\left(0\right)+E\left(0\right)+I\left(0\right)\right)\\ \; & \leq t^{\alpha }E_{\alpha ,\alpha +1}\left(-\mu t^{\alpha }\right)\mu +E_{\alpha ,1}\left(-\mu t^{\alpha }\right)=1 \end{array}$$

by Equation (7). □

**Proof of Lemma** **2.**
*For the local stability of a disease-free equilibrium, we must evaluate the Jacobian matrix at* DFE≡(1,0,0)

$$J\left(DFE\right)=\left[\begin{array}{ccc} -\mu & 0 & -\beta \\ 0 & -(\mu +\delta ) & \beta \\ 0 & \delta & -(\mu +\sigma ) \end{array}\right]$$

The eigenvalues of the matrix *J* are,

$$\lambda_{1}=-\mu ,$$

$$\lambda_{2}=\frac{-\left(\delta +2\mu +\sigma \right)-\sqrt{\Delta }}{2},$$

$$\lambda_{3}=\frac{-\left(\delta +2\mu +\sigma \right)+\sqrt{\Delta }}{2},$$

where $\delta =\delta^{2}+4\delta \beta -2\delta \sigma +\sigma^{2}$. From this it is clear that $\lambda_{1}$ is negative and since

$$\Delta =\delta^{2}+4\delta \beta -2\delta \sigma +\sigma^{2}={\left(\delta -\sigma \right)}^{2}+4\delta \beta >0$$

then $\lambda_{2}$ and $\lambda_{3}$ are real-valued numbers. Hence $\lambda_{2}<0$. But, $\lambda_{3}<0$ is true when

$$\frac{-\left(\delta +2\mu +\sigma \right)+\sqrt{\delta^{2}+4\delta \beta -2\delta \sigma +\sigma^{2}}}{2}<0$$

which is equivalent to βδ<(μ+σ)(μ+δ), proving the first part. The Jacobian matrix calculated at EE is given by

$$J\left(EE\right)=\left[\begin{array}{ccc} -\mu R_{0} & 0 & -\beta \frac{1}{R_{0}}\\ \mu (R_{0}-1) & -(\mu +\delta ) & \beta \frac{1}{R_{0}}\\ 0 & \delta & -(\mu +\sigma ) \end{array}\right]$$

which has a characteristic polynomial,

$$-\lambda^{3}-\lambda^{2}\left[\left(\mu +\delta \right)+\left(\mu +\sigma \right)+\mu R_{0}\right]-\lambda \left[\mu R_{0}\left(2\mu +\delta +\sigma \right)\right]+\mu \left(R_{0}-1\right)\left(\mu +\sigma \right)\left(\mu +\delta \right).$$

Because that polynomial has a degree of 3, we choose to test the Routh-Hurwitz conditions to see if EE is stable.

$$a_{1}=\mu R_{0}+\left(2\mu +\delta +\sigma \right)>0$$

$$a_{3}=\mu \left(R_{0}-1\right)\left(\mu +\sigma \right)\left(\mu +\delta \right)>0$$

With these conditions we check that the determinant, $D_{2}>0$.

$$D_{2}=a_{1}a_{2}-a_{3}=\left(\mu R_{0}+2\mu +\delta +\sigma \right)\left(\mu R_{0}\left(2\mu +\delta +\sigma \right)\right)-\left(\mu \left(R_{0}-1\right)\left(\mu +\sigma \right)\left(\mu +\delta \right)\right)$$

$$=\mu [\mu R_{0}^{2}\left(2\mu +\delta +\sigma \right)+{\left(2\mu +\delta +\sigma \right)}^{2}R_{0}-R_{0}\left(\mu +\sigma \right)\left(\mu +\delta \right)+\left(\mu +\sigma \right)\left(\mu +\delta \right)]$$

$$=\mu [\mu R_{0}^{2}\left(2\mu +\delta +\sigma \right)+{\left(\mu +\sigma \right)}^{2}R_{0}+{\left(\mu +\delta \right)}^{2}R_{0}+\left(\mu +\sigma \right)\left(\mu +\delta \right)R_{0}+\left(\mu +\sigma \right)\left(\mu +\delta \right)]>0$$

From this, all Routh-Hurwitz conditions are met and all the eigenvalues of the Jacobian matrix at EE are negative, meaning that $Re(\lambda_{k})<0,$k=1,2,3. □

## Appendix B. Data Sets and Parameter Estimation

### Appendix B.1. New York

Monthly reported infections of measles from September 1961 to January 1963 in New York City, USA are given in Table A1. Parameter estimation of New York’s measles data from September 1961 to January 1963 using both ODE model and FDE model. The estimated parameters values for the classical ODE model are $\left(\mu ,\beta ,\delta ,\sigma \right)=$(0.0028,119.22,16.73,10.19) with the sum of square error, SSE = 1.29 × $10^{6}$ and for the FDE model are $\left(\alpha ,\mu ,\beta ,\delta ,\sigma \right)=\left(0.999999999524,0.0029,116.34,19.39,10.37\right)$ with the sum of square error, $SSE=1.34\times 10^{6}$.

**Table A1:** Reported infections of measles from September 1961 to January 1963 in New York, USA [57].

Year	Months	Cases	Year	Months	Cases	Year	Months	Cases
1961	September	109	1962	March	5839	1962	September	58
1961	October	123	1962	April	7875	1962	October	86
1961	November	383	1962	May	6555	1962	November	125
1961	December	1043	1962	June	2866	1962	December	145
1962	January	1725	1962	July	1075	1963	January	184
1962	February	3056	1962	August	266			

### Appendix B.2. London

Biweekly reported infections of measles in 1961 in London, United Kingdom are given in Table A2. Parameter estimation of measles Portsmouth data in 1961 using both ODE model and FDE model. The estimated parameters values for the classical ODE model are $\left(\mu ,\beta ,\delta ,\sigma \right)$ = $\left(6.79\times 10^{-4},153.44,1.99,4.27\right)$ with the sum of square error, $SSE=2.01\times 10^{6}$ and for the FDE model are $\left(\alpha ,\mu ,\beta ,\delta ,\sigma \right)=\left(0.999999999614,0.0015,122.68,2.18,8.75\right)$ with the sum of square error, $SSE=2.04\times 10^{6}$.

**Table A2:** Biweekly reported measles infections in 1961 in London, UK [57].

**weeks**	0	2	4	6	8	10	12	14	16	18	20	22	24	26
**cases**	1636	2700	2639	4805	6543	6389	5545	5374	4272	2322	1810	1409	1037	767
**weeks**	28	30	32	34	36	38	40	42	44	46	48	50	52	
**cases**	514	375	265	199	121	86	76	89	87	73	70	59	45	

### Appendix B.3. Portsmouth

Biweekly reported infections of measles in 1961 in Portsmouth, United Kingdom are given in Table A3. Parameter estimation of measles Portsmouth data in 1961 using both ODE model and FDE model. The estimated parameters values for the classical ODE model are $\left(\mu ,\beta ,\delta ,\sigma \right)=\left(10^{-6},228.61,0.46,3.33\right)$ with the sum of square error, $SSE=4.57\times 10^{4}$ and for the FDE model are $\left(\alpha ,\mu ,\beta ,\delta ,\sigma \right)$ = (0.875784505546, $2.56\times 10^{-4}$, 278.72, 1.52,5.24) with the sum of square error, $SSE=3.22\times 10^{4}$.

**Table A3:** Biweekly reported infections of measles in 1961 in Portsmouth, UK [57].

**weeks**	2	4	6	8	10	12	14	16	18	20	22	24	26	28	30
**cases**	4	30	58	174	310	407	640	847	555	523	337	242	144	91	29
**weeks**	32	34	36	38	40	42	44	46	48	50	52				
**cases**	21	25	28	13	5	2	1	2	0	2	0				

### Appendix B.4. Parameter Estimations

**Table A4:** Parameter estimation and statistical measures of fittings for the classical ODE model and FDE model using different data sets.

Data	Model	Estimated Parameters, (α,μ,β,δ,σ)	SSE	AIC	BIC
New York	ODE	(NA,0.0028,119.22,16.73,10.19)	$1.29\times 10^{6}$	250.54	253.87
	FDE	(0.999999999524,0.0029,116.34,19.39,10.37)	$1.34\times 10^{6}$	255.36	259.53
London	ODE	$\left(NA,6.79\times 10^{-4},153.44,1.99,4.27\right)$	$2.01\times 10^{6}$	389.36	394.54
	FDE	(0.999999999614,0.0015,122.6849,2.18,8.75)	$2.04\times 10^{6}$	393.34	398.85
Portsmouth	ODE	$\left(NA,10^{-6},228.61,0.46,3.33\right)$	$4.57\times 10^{4}$	277.94	282.98
	FDE	$\left(0.875784505546,2.52\times 10^{-4},278.72,1.52,5.24\right)$	$3.22\times 10^{4}$	271.92	278.21
